# Ethnobotanical notes about some uses of medicinal plants in Alto Tirreno Cosentino area (Calabria, Southern Italy)

**DOI:** 10.1186/1746-4269-3-34

**Published:** 2007-11-05

**Authors:** Maria Lucia Leporatti, Massimo Impieri

**Affiliations:** 1 Dipartimento di Biologia Vegetale, Università "La Sapienza" P. le A. Moro, 5- Roma, Italia

## Abstract

**Background:**

The present paper contributes to enrich the ethnobotanical knowledge of Calabria region (Southern Italy). Research was carried out in Alto Tirreno Cosentino, a small area lying between the Tyrrhenian coast and the Pollino National Park. In the area studied medicinal plants still play a small role among farmers, shepherds and other people who live far from villages and built-up areas.

**Methods:**

Information was collected by interviewing native people, mainly elderly – engaged in farming and stock-raising activities – and housewives. The plants collected, indicated by the locals, have been identified according to "Flora d'Italia". The *exsiccata *vouchers are preserved in the authors' own *herbaria*.

**Results:**

52 medicinal species belonging to 35 families are listed in this article. The family, botanical and vernacular name, part of the plant used and respective manipulation are reported there and, when present, similar or identical uses in different parts of Calabria or other Italian regions are also indicated.

**Conclusion:**

*Labiatae*, *Rosaceae *and *Leguminosae *are the families most frequently present, whilst *Compositae *and *Brassicaceae *are almost absent. The uses of the recorded species relate to minor ailments, mainly those of the skin (15 species), respiratory apparatus diseases (11), toothache, decay etc. (10) and rheumatic pains (8). The easy availability of these remedies provides a quick way of curing various minor complaints such as tooth-ache, belly and rheumatic pain and headaches and can also serve as first aid as cicatrizing, lenitive, haemostatic agents etc. The role in veterinary medicine is, on the contrary, more important: sores, ulcers, tinea, dermatitis, gangrenous wounds of cattle, and even respiratory ailments are usually cured by resort to plants.

## Background

**Figure 1:**
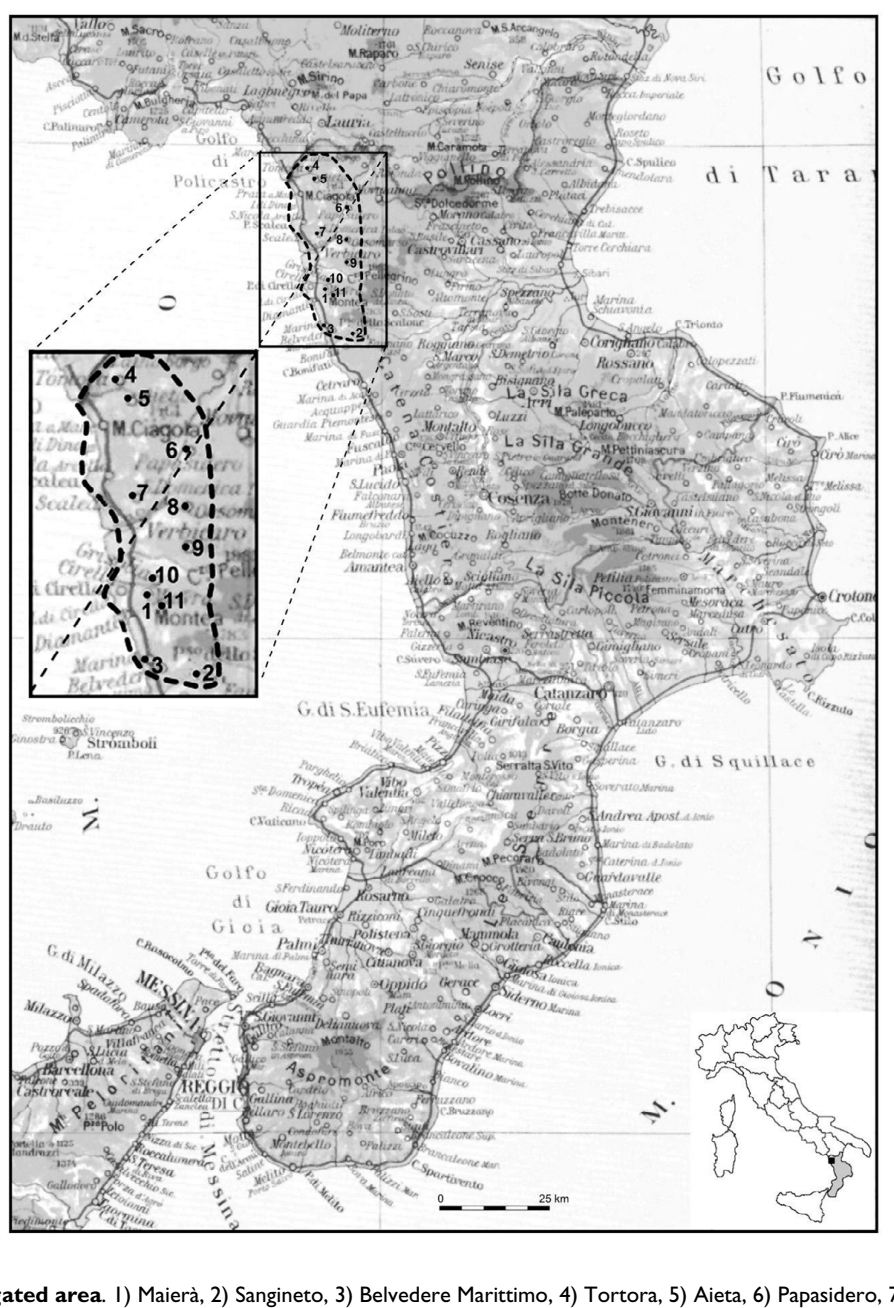
**Map of investigated area**. 1) Maierà, 2) Sangineto, 3) Belvedere Marittimo, 4) Tortora, 5) Aieta, 6) Papasidero, 7) S. Domenica Talao, 8) Orsomarso, 9) Verbicaro,10) Grisolia, 11) Buonvicino.

Calabria, from the ethnobotanical point of view, is one of the least investigated of the Italian regions, with the exception of few contributions by Barone [1], Leporatti and Pavesi [2], Bernardo [3], Lupia [4], Passalacqua *et al*. [5,6]. This paper makes a short contribution to the knowledge of popular medicine founded on traditional uses of plants. This research is focused on a limited area: Alto Tirreno Cosentino, that administratively comes under the province of Cosenza. This area is bordered to the north by the region of Basilicata, to the east by the coastal belt of the Tyrrhenian sea, from the mouth of the Noce river to the mouth of the Sangineto in the south, whilst the western includes the slopes of the Apennine coastal chain located within the Pollino National Park [Fig. 1]. The climate is typically Mediterranean, with dry summers, rainy and windy winters. Rainfall is around 700–1000 mm/year.

The area under consideration, rises from the coastal belt to a height of 2000 m asl and, consequently, the flora and vegetation present different typologies: in the coastal area where a shrubby vegetation with evergreen and termophilous species such as *Phillyrea latifolia *L., *Myrtus communis *L.*, Pistacia lentiscus *L. and *Quercus ilex *L., etc. dominate, alternating with areas where mainly *Gramineae *and xerophilous annuals grow. In the mountainous zone, mesophilous woody species are mainly present i.e. *Fagus sylvatica *L.*, Acer psdeudoplatanus *L., *Tilia platyphyllos *Scop., *Ilex aquifolium *L., etc.

Until the first half of the last century, people living in this area were engaged in agricultural activities, and the medicinal plants gathered and traded played a truly important role in the local economy (SVIMEZ [7]). Over time people started to leave the countryside and agricultural activities so today little of this knowledge and pertinent uses survives, and that only amongst farmers, shepherds and elderly people.

## Methods

The research, that started in December 2005 and ended in September 2006, was conducted in several villages or near the coastal areas such as Belvedere Marittimo, whereas Tortora, Aieta, Papasidero, S. Domenica Talao, Orsomarso, Verbicaro, Grisolia, Maierà, Buonvicino and Sangineto running north to south, all fall into the inland part of the area studied.

Fifty informants were involved in the interviews, mainly farmers, shepherds and housewives, native to the area or having lived there since childhood, the most of them over 65 years old.

Special care was taken in collecting information to avoid any unoriginal information which, probably influenced by sources such as books, magazines etc., were rejected. Interpretation or translation into technical or medicinal terms of the information received was carefully avoided so as to have a true picture of customs and uses. The information was usually imparted in the local dialect, and plants were indicated with vernacular names. The informants were aware of the aims of the research and the end use of the information they gave. They also collaborated in collecting and recognising plants. Moreover, they also indicated also where plants were easily available. That is to say, informants sometimes indicated precise plants i.e. *Parietaria officinalis *L. or *Arum maculatum *L. which are quite rare compared to others of the same genera. They probably cannot distinguish the different species when these are very similar so, in this paper, these are reported taken into consideration the fact that therapeutic uses could also involve the less rare species. After having collected the species, the *exsiccata *were prepared and then identified according to the nomenclature of Flora d'Italia by Pignatti [8]. In several cases also "An annotated list of the Italian Vascular Flora" (Conti *et al*. [9]) was consulted. The voucher specimens of *exsiccata *have been preserved in the authors' own *Herbaria*.

The recorded data were compared with specific ethnobotanical literature, not limited only to central and southern Italian regions, in order to identify analogies, differences or eventual uses not cited previously: Arcidiacono and Pavone [10], Basile *et al*.[11], Bellomaria and Della Mora [12], Bruno *et al*. [13], Caneva *et al*. [14], Corrain and Zampini [15], Corsi *et al*. [16,17], De Feo *et al*. [18], De Feo and Senatore [19], Famiglietti *et al*. [20], Gastaldo [21], Guarrera [22,23], Lentini *et al*. [24-28], Lentini and Raimondo [29], Leporatti *et al*. [30,31], Leporatti and Corradi [32], Lodi [33]**, **Lomazzi [34], Longo [35], Maccioni and Marchini [36,37], Manzi [38], Pieroni [39], Pieroni *et al*. [40], Raimondo and Lentini [41] Renzetti and Tajani [42], Tammaro [43], Tammaro and Pietrocola, [44], Viegi *et al.*[45], Viegi *et al*. [46], Zampiva [47], Guarrera[48].

## Results

In the additional file: 1, 52 species belonging to 35 families are listed. Family, botanical and vernacular name, part of the plant used and respective manipulation are reported; in addition the locality (in abbreviated form) where information were collected. In the last column of additional file: 1 are highlighted, when present, the similar or identical uses in Calabria or in other Italian regions. Veterinary uses are highlighted in rimmed bold boxes. Both families and species, are listed in alphabetical order for ease of consultation.

## Discussion and Conclusion

From this short survey it is possible to draw some considerations: the knowledge of phytotherapeutic practice is not homogeneous in the area examined but, more or less, crops up particularly in the inland villages, linked to ancient customs and to agricultural, stock-raising and woodland activities. Phytotherapy was not, in fact regarded as a general patrimony, but was considered somewhat as a personal secret since in the past, healers did not reveal their knowledge which was their source of gain. This scanty and residual knowledge is highlighted in such a way (apart from in the SVIMEZ report [7]) also by the data quoted in the previous ethnobotanical papers concerning Calabria [1,2,5,6] – pointed out in the last column of additional file: 1. Many of these species have several uses. Also significant is the equality – or in some cases even the lack – of vernacular names (with the exception of six plants: *Adiantum capillus-veneris, Helleborus foetidus, Malus domestica, Rosa canina *and *Rubus ulmifolis*) in the coastal and inland villages. In the past, the coastal area was deserted as, threatened by invasion by Saracens or Byzantines, or by Norman or Spanish domination, the people took shelter in the interior. It was only in the second half of the 19^th ^century that this trend was inverted with people abandoning the countryside and tourism developing along the coast. Therefore, knowledge pertaining to phytotherapy or to medicinal plants in general in the coastal villages must be related, where it has not been lost, to that of the interior. It is clear that folk phytotherapy today is greatly reduced and largely abandoned, swept away by pharmaceutical technology. What remains of this centuries old knowledge relates mainly to minor diseases and ailments such as those concerning the skin, cicatrising, lenitive and haemostatic agents, treatment of warts and corns (15 species) as well as the range of rheumatic pains (8 species). These remedies offer relief in cases of respiratory tract ailments such as colds, coughs and even bronchitis (11 species). Toothache, decay and gingivitis are also there treated (10 species); whilst cures for severe diseases of other parts of the body are less frequent and almost entirely abandoned. There are, however, some exceptions:*Zea mays *as a diuretic and lenitive for renal complaints, *Urtica dioica *L. (and close species) used to protect and treat the liver and kidneys, treating piles and as an edible plant. The case of *Spartium junceum *L. is also interesting, considered to be an antineoplastic in mixture with *Cynodon dactylon *L. and *Arundo donax *L. (the informant said precisely "......*in the event of tumour*...." without specifying which kind of tumour). It is important to remember that *Spartium junceum*, in spite of the presence of sparteine, isosparteine and citisine alkaloids, is still used in different areas and, here as elsewhere, toxic plants here are used without any particular concern in Italy. The root of *Spartium junceum *is believed to expel kidney gall in the Calabria and Marche regions [30]; [23] and the infusion of ash from its flowers and branches is considered diuretic and sedative in cases of gastralgia [2].

Other toxic plants are used: *Ruta graveolens *L. here employed only for conjunctivitis ("gust of wind"), headache and as an antihelmintic, but it is also well known in Calabria and several other Italian regions in relation to a great range of illnesses [47]. In spite of its toxicity medicinal properties of this species have been claimed since the time of *Schola Salernitana *(1100 A.D.)*" Nobilis est Ruta quae lumina reddit acuta et e pulcibus fac loca tuta" *and still today several regional proverbs refer to its numerous medicinal activities. "*Ruta sette mali stuta*" that is to say "*Ruta *dispels (at least) seven diseases".

The toxic *Polypodium vulgare *considered useful in renal complaints for kidney galls, has the same use in the Abruzzo region [32]. The powdered root of the highly toxic *Atropa belladonna *is used as an anti-rheumatic and anti-arthritic also in Abruzzo [43].

This similarity in the therapeutic uses of plants, both in Calabria and in different Italian regions seems to furnish important corroboration of the validity the remedies reported

Concerning the families it is possible to observe that *Labiatae *(5 species), *Poaceae *(5 species) and *Rosaceae *(4 species) are the most frequently employed, whilst it is surprising that several families such as,*Umbelliferae, Cruciferae *and even *Compositae *traditionally well represented in Italian folk phytotherapy given their wealth of medicinal plants, are here scarcely considered or absent at all, such as *Liliaceae*. Nevertheless it must not forget that still today there is frequent resort to a number of homemade syrups, prepared as decoctions, obtained by mixing many different species:*Malva sylvestris, Tussilago farfara, Sisymbrium officinale, Tilia *sp. pl.*. Althaea officinalis, Mentha pulegium*,* Ficus carica, Hordeum vulgare, Cynodon dactylon, Thymus pulegioides*, etc. These syrups, prepared according to different recipes and customs which vary from village to village, include the use of some cultivated species i.e.: *Prunus persica, Ficus carica, Malus domestica *etc. The syrups are used indifferently for therapeutic purposes in several ailments: coughs, colds, stomach aches or as mild sedatives, but without any explanations of the role played by the single plant, very probably added to correct or sweeten the taste. On the contrary veterinary medicine still seems to be alive and well, and practiced in daily life (veterinary uses are highlighted in italics, in the boxes within additional file: 1). This is easy to understand since shepherds and farmers living with their herds, flocks, cattle or poultry, far from the villages or built-up areas, are forced to use remedies immediately available in the fields and their kitchen gardens. Moreover it is important to remember that many of these phytotherapic uses, experimented over a long period of time, have parallels in numerous parts of Italy where they are not only present but are prepared in the same manner. For example, *Helleborus foetidus *L., in spite of its toxicity, is used in cases of bronchitis and pneumonia in cattle also in Emilia Romagna [34], Trentino [42], and Latium regions [22]. A decoction of *Lupinus albus *is used as a wash to treat dermatitis in cattle in Marche[12], Toscana 16], and Abruzzo [38]. Sores and ulcers caused by pack saddle can be healed by the ash of the stems of *Triticum aestivum *mixed with olive oil and the same use is also recorded in other Italian regions (Gastaldo [21]), where the stimulating and cicatrizing properties of phytostimuline are well known.

This can also explain why certain human complaints are treated with plants rather than others: e.g. *Plantago *spp. leaves are a good detergent for small wounds, and the hairy leaves of *Verbascum *spp. or of *Sideritis syriaca *stop bleeding caused by cuts. According to recent studies, the similar species of *Sideritis *also shows good antibacterial activity [11]. Colds, coughs, fevers and even bronchitis can be treated immediately with *Tussilago farfara*, *Rosa canina *and *Mentha pulegium*, whilst a sudden tooth-ache is soothed with *Malva sylvestris *and the exudates from the branches of *Pistacia lentiscus *is a good mouth-wash for inflammation of the mouth cavity. Rheumatic pains, affecting mainly elderly people, can be relieved by a decoction or infusion of *Salix alba *bark or *Agave americana *leaves (this last frequently cultivated) and, in spite of its toxicity, even the dried root of *Atropa belladonna*. All of these species are well known, and have parallel uses in many Italian regions. It should be noted that *Diospyros kaki*, *Agave americana*, and *Eucaliptus globulus *are the only exotic plants present in this repertory. The first has mainly an alimentary employ; *Agave americana*, and *Eucaliptus globulus *are widespread used in Italian folk medicine [47] and their uses are both well justified; the former by the presence in the leaves and rhizome of a saponin, revulsive when used externally and the latter is used as an antiseptic for the respiratory tract, by the high content of essential oils, long used also in pharmaceutical preparations.

In conclusion it is possible to point out that, despite the vast array of current pharmaceutical technology, what remains of ancient folk phytotherapy survives and still plays a role given when the ready availability of the ingredients required, the simplicity of the preparations (infusion, decoction, etc.) and the fact that it can make daily life easier.

## Supplementary Material

Additional file 1 Medicinal plants in Alto Tirreno Cosentino area (Calabria, Southern Italy) Click here for file
