# Supplementary material for: Ethnobotanical notes about some uses of medicinal plants in Alto Tirreno Cosentino area (Calabria, Southern Italy)
Source: J Ethnobiol Ethnomed. 2007 Nov 5;3:34. doi: 10.1186/1746-4269-3-34 (PMC2222047; doi:10.1186/1746-4269-3-34)
Supplement: Additional file 1 — Medicinal plants in Alto Tirreno Cosentino area (Calabria, Southern Italy) [file 1746-4269-3-34-S1.pdf]

Additional file 1. Folk medicinal plant uses in Alto Tirreno Cosentino area (Calabria, Southern Italy)

Scientific name/ Family	*Vernacular name*	*Folk medical use*	*Part(s) used*	*Manipulation*	*Sites where the folk medical use has been recorded*	*Notes about folk uses in different areas of Calabria [1, 2, 5, 6] and/or in other Italian regions [7- 48]*
1 Agavaceae						
Agave americana L.<br>ga 1	Aloia	Muscular and rheumatic pains	Leaves	The decoction, left overnight, is locally rubbed	Verb. Ma.	
2 Anacardiaceae						
Pistacia lentiscus L.<br>ana 1	Scinu	Swollen gums	Branches	The sap dropping<br>out after having cut the stems, in mouth-washes	Verb., Ma.<br>Belv.<br>M. mo	The same use has been recorded in different areas of Calabria region [7]
3 Araceae						
Arum maculatum<br>L.<br>ara 1	Anzana	Antiseptic,<br>Cicatrizing,<br>To treat corns	Tuber<br>Leaves	The sliced tuber or the fresh leaves are boiled and locally applied on wounds and burns.<br>The juice from the<br>squashed tuber is<br>externally applied<br>on warts and corns	Verb.	The informants indicated this species, but probably *Arum italicum* Miller is also used for the same purposes
4 Araliaceae						
Hedera helix<br>L.<br>arali 1	Giss'	To treat toothache<br>and decay	Stems and branches	The sap dropping<br>out after having cut the stem is locally applied	Verb., Gri.,	In the province of Reggio Calabria this species is used to soothe cough, bronchitis, and to heal sores [6]
5 Betulaceae						
Alnus cordata L.<br>betu 1	Avezen'	To remove warts and corns	Leaves	The fresh leaves are locally rubbed	Ma. Gri.,<br>Papa.	
6 Caprifoliaceae						
Sambucus ebulus<br>L.<br>capri 1	Jemili	*To treat lice in poultry*	*The whole plant*	*A bunch of the fresh plants is hung up inside the hen-house*	*Verb., Gri.*	*The same use has been recorded in Veneto region [47]*
Sambucus nigra<br>L.<br>capri 2	Majo	To treat swollen breast and legs, and rheumatic pains	Dried flowers	Decoction in external washes on the affected body district	Buonv.,<br>Papa.	In Calabria other uses of this species have been recorded: the decoction of flowers with sugar is considered expectorant (without sugar is a lenitive in case of erythemas) and an ointment obtained from the bark or the fresh leaves is used against burns.<br>The cataplasm made of

						the heated leaves was considered anti-rheumatic; it is also applied on the breast to resolve milk obstruction.<br>The infusion of the flowers is considered a good mouthwash for treating inflammations and thrush in children, or eye salve in conjunctivitis [1]. It was used also as an anti-rheumatic and purgative [6]
7 Cesalpinaceae						
Ceratonia siliqua L.<br>cesa 1	Caroba	Emollient	Fruits	Decoction with *Ficus carica* L, *Malva sylvestris* L., and *Prunus persica* (L.) Batsch	Verb.	
8 Compositae						
Matricaria chamomilla L.<br>aste 1	Capomilla	To treat abdominal pains	Flowers	Decoction with *Malva sylvestris* L., *Calamintha nepeta* (L.) Savi, and *Sambucus nigra* L.	Verb. Gri. Buonv,. Papa.	The plant in other districts of Calabria is used as a digestive, for treating cough, and as a mild sedative [6]
Tussilago farfara L.<br>aste 2	Frunn' i canale	Against cough, balsamic	Roots, leaves and flowers	Syrup prepared by a decoction of the roots, or infusion of the fresh leaves and flowers mixed with *Malva sylvestris* L., *Hordeum vulgare* L., and *Urtica* spp.	Verb., Papa	The species is also used in the region as a diuretic and for treating furunculosis [1]
9 Crassulaceae						
Umbilicus rupestris (Salisb.) Dandy<br>crassu 1	Cucch'	To treat chilblains, warts	Leaves without outer "peel"	Cataplasm or bandage imbibed of the decoction, locally applied	Orso. Sang.	The same medical claim has been made using the heated leaves [6]
10 Cruciferae						
Sisymbrium officinale L.<br>brassi 1	Erisimo	To treat cold	Whole plant	Decoction with *Tilia platyphyllos* Scop. and *Salix alba* L.	Verb., Sang.	
11 Ebenaceae						
Diospyros kaki L. Fil.<br>ebe 1	Cachino	As a tea substitute	Leaves	Infusion	Aie.	
12 Equisetaceae						
Equisetum telmateja Ehrh.<br>equi 1	Cuda ' i vurpa	Haemostatic Stomachic	Stems	Compresses imbibed of the infusion	Buonv.	Toxic plant!
13						

Euphorbiaceae						
Mercurialis annua L. eupho 1	Mercurella	Laxative Diuretic	Fresh plant before flowering	Decoction.	Verb.	The decoction has been recorded as a laxative for cattle in Campania ([19, 41] and as a depurative in Liguria [37]
		To aid calves in suckling		*Decoction mixed with* Matricaria chamomilla *L.*		
14 Fagaceae						
Quercus sp. faga 1	Cerza	Intestinal astringent, detoxifying<br><br>Cicatrizing	Dried bark of young stems and galls	Decoction (internally)<br><br>Decoction, in external washes	Torto.	The informants indicated *Quercus robur* L., but probably also other species of the genus were equally used.<br>In different districts of Calabria region *Q. frainetto* Ten. is used for treating acne, rheumatisms, and for healing burns [2]
15 Gramineae						
Arundo donax L. poa 1	Canna	To treat throat inflammations and bronchitis (see also *Spartium junceum* L.)	Stems when they are still green<br><br>Rhizome	Stems filled with sugar and heated on fire until they melt, then ingested<br><br>Decoction with *Cynodon dactylon* L. rhizomes	Verb.	The internal membrane of a dried stem from this species is applied on wounds as a haemostatic and cicatrizing mean.<br>The sap mixed with sugar has been recorded as a mean for treating whooping cough [6]
Avena sativa L. poa 2	not reported	To treat abdominal pains	Seeds	Decoction with *Malus domestica,* (Bork.) Bork., *Hordeum vulgare* L., and *Ficus carica* L. (with honey)	Verb	
Cynodon dactylon L. poa 3	Gramigna	Diuretic	Seeds	Decoction	Verb.	Decoction of the rhizome is considered able to heal cystitis; it is also lenitive for digestive, urinary and genital inflammations and it is a mild laxative too.<br>The decoction of the aerial parts is considered an anti-rheumatic. [6]
Hordeum vulgare L. poa 4	Oriu	To treat cold and abdominal pains	Seeds	Decoction with sliced *Malus domestica* (Bork.) Bork. fruits and *Ficus*	Verb., Papa.	A prolonged decoction of the seeds has been recorded as a mean for treating throat inflammations (gargles).

				carica L. (with honey)		A poultice of the same plant is considered lenitive on the skin and able to cure head whitlows [6]
Triticum aestivum L. poa 5	Granu	*To treat wounds and sores in cattle (e.g. caused by pack-saddle)*	*Stem*	*The ash from the stems is mixed with olive oil, then locally applied*	*Verb.*	*Grinded seeds have been recorded to be put on the skin as an anti-diaphoretic in Val di Cècina (Toscana) [45]*
		To treat finger bones dislocation	Bran	Cataplasm with bran mixed with boiled vinegar		
Zea mays L. poa 6	Migliu	To heal cystitis and inflammations of the urinary tract	Stigmata	Decoction	Verb.	
16 Juglandaceae						
Juglans regia L jugla 1	Nucia	Against excessive feet perspiration	Fresh leaves	Leaves put into the shoes	Orso. Verb.	Similar uses have been recorded elsewehere: the juice of leaves is said to be able to strengthen the skin of the feet and to preserve them from ulcers and sores [1]. The same species has been also recorded for healing ulcers [2] and as an anti-rheumatic [1]
17 Labiatae						
Calamintha nepeta L. lami 1	Niepita	*Against rhagades and mastitis of cattle*	*Whole plant (dried or fresh)*	*Decoction for local washing*	*Ma. Gri., Verb.*	*The decoction has been recorded as a mean against lice in the Majella area (Abruzzo) [48]*
		To treat bee and insects stings and as an anti-haemorrhagic	Flowering tops	The fresh plant rubbed on the skin. Infusion		
Mentha pulegium lami 2	Righinedda	To treat cough, inflammations of the respiratory tract, and cold	Whole plant	Infusion	Orso. Verb.	
Salvia officinalis L. lami 3	Salivia	To treat swollen testicles and related pains (also in presence of bleeding)	Leaves	Decoction to drink	Orso.	Infusion of the leaves has been used against cough, asthma, and as a digestive. Compresses made of the decoction has been considered useful for treating wounds and sores [6]
Sideritis syriaca L. lami 4	Erva du taglio	Haemostatic for little wounds	Leaves	Fresh leaves directly applied on wounds	Verb.	

Thymus pulegioides L<br>lami 5	not reported	Cough, cold	Leaves, flowers	Decoction with *Sisymbrium officinale* L., *Tilia platyphyllos* Scop., *Salix alba* L., *Eucaliptus globulus* Labill., and *Tussilago farfara* L.	Verb., Sang.	
18 Leguminosae				.		
Lupinus albus L<br>faba 1	Luppino	*To treat dermatitis in cattle*	*Whole plant*	*Decoction in external washes*	*Gri.*	*Against lice and ticks the same plant has been reported in diverse Italian areas: Marche [12], Toscana [16] (macerates of the seeds in water), and Polesine (infusions) [15].*<br>*The infusion of this species is also applied on horn and ears in Abruzzo [38]*
Spartium junceum L.<br>faba 2	Sparto	In event of tumour	Root	Decoction with *Cynodon dactylon* L. rhizomes and *Arundo donax* L.	Verb.	The same species has been recorded to be used against stomach-aches [2], as a diuretic [2, 6] and for treating kidney stones and viper bites [6]
19 Malvaceae						
Althaea officinalis L.<br>malva 1	not reported	Cough, cold	Roots	Decoction with *Tussilago farfara* L., *Eucalyptus globulus* Labill., and *Malva sylvestris* L.	Verb.	
Malva sylvestris L.<br>malva 2	Mariva	Lenitive, to treat gingivitis	Leaves and flowers	Infusion or decoction, alone or with *Matricaria chamomilla* L.	Ma. Verb. Aie., Papa.	Decoctions of leaves and flowers have been recorded to be used as cicatrizing means; cataplasms mixed with sugar have been used on boils and in case of whitlows. A decoction of the flowers has been recorded to treat diseases of the respiratory tract; fumigations have been recorded in case of ear pains, and the flowers as antispasmodic means in abdominal pains [1]
20 Moraceae						
Ficus carica L.<br>mora 1	Figu	Co-adjuvant in the therapies for	Dried or fresh	Decoction with *Malus*	Verb.	

		treating cold, flu, and cough	fruits	*domestica* (Bork.) Bork., *Hordeum vulgare* L.. *Avena sativa* L. and honey		
21 Myrtaceae						
Eucalyptus globulus Labill. myrta 1	Calipto	Antiseptic of the respiratory tract	Leaves	Decoction with *Tussilago farfara* L., *Malva sylvestris* L., and *Althaea officinalis* L.	Verb.	The decoction of the leaves and the bark has been considered useful for malaria and jaundice. Macerates in alcohol were locally applied as an anti-rheumatic. The bark mixed with leaves of *Quercus* sp., in decoctions, was employed in cases of congested spleen [1]. Decoction of leaves or boiled leaves were locally applied to wash little wounds and cuts [2]
Myrtus communis L. myrt a 2	Murtidda	To treat cold<br><br>Against excessive sweating of the feet	Leaves<br><br>Ash from leaves	Infusion<br><br>The internal part of the shoes were sprinkled with ash	S.Dom. Tal., Verb	Leaves, burnt and powdered, are sprinkled on reddened skin of children. [1]. A decoction of the leaves was used in footbaths [2].
22 Oleaceae						
Ligustrum vulgare L. ole 1	Vuccàina	*Cattle stomatitis, chaps out or inside mouth (mainly in sheep and goats)*	*Leaves*	*Decoction as mouthwash or externally applied*	*Gri., Verb.*	
23 Plantaginaceae						
Plantago lanceolata L. planta 1	Cincu nirvi	Cicatrizing<br><br>To treat bruises finger bruises	Fresh leaves	Squashed leaves locally applied<br><br>As bandage	Verb., Gri. Sang.	The same species has been recorded to stop milk secretion in cattle in Liguria [37], as haemostatic and cicatrizing in Toscana (both for cattle and humans, [16, 45]) and in Abruzzo [43]

Plantago major L.<br>planta 2	Cintu nirvi	Anti-helmintic<br><br>Detergent for wounds	Leaves.	Juice from squashed leaves; drunk<br><br>Juice f from squashed leaves, in external washes	Ma., Verb.	The same use has been recorded in Abruzzo and in Toscana [43, 17] .<br>The plant has been quoted also fro treating head whitlows and furuncles [1, 6], whilst crushed leaves are locally applied on wounds [6]
24<br>Polypodiaceae						
Adiantum capillus-veneris<br>polyp 1	Filicieddu, Capiddu-vennari	To treat dental neuralgia and otitis	Leaves	Decoction	Orso.	Decoctions of the leaves have been claimed to be able to regulate the female cycle; mixed with other species, this plant has been recorded as a diuretic, antispasmodic, and reliefing pains due to kidney stones [1].<br>Toxic plant!
Polypodium vulgare L.<br>polyp 2 and *Polypodium interjectum* Shivas<br>polyp 3	Filici	To treat kidney stones and dysfunctions of the urinary tact	Leaves	Decoction	Verb.	The ash arising from the leaves - mixed with olive oil - is locally applied on severe burns [6].<br>Toxic plant!
25 Primulaceae						
Cyclamen sp.pl.	Not reported	Against warts and corns	Fresh tuber	The poultice from the tuber, locally applied	Verb.	Toxic plant!
26<br>Ranunculaceae						
Helleborus foetidus L.<br>ranu 1	Erva da rugna, Ricchiaina	*Against cattle tinea*<br><br>*To treat bronchitis and pneumonia of cattle*	*Fresh leaves*	*Rubbed on the skin*<br><br>*Bits of the root must be inserted in a fitting hole in the ear*	*Ma., Verb.*	*The same use has been recorded in Latium [22], in Emilia Romagna [34], and in Trentino [42]. This use is largely widespread in Calabria [5] and also in Emilia Romagna [33]*
						The use of this plant, although very toxic, as an antiodontalgic in human therapy has been reported in Calabria [1, 2]
27 Rhamnaceae						
Ziziphus jujuba Miller<br>rhamna 1	Jujolo	Cough	Ripe fruits	Decoction with *Ficus carica* L., *Malus domestica* Bork., *Hordeum vulgare* L., *Avena sativa* L., and honey	Verb.	
28 Rosaceae						
Malus domestica	Pirajna,	Sweetener	Fruits	Sliced fruits in	Verb.	Decoctions in milk of

Bork.<br>ro 1	Milu			several home-made syrups		the flowers and the leaves are said to be lenitive in cough, anti-fever, and sedative [6]
Prunus persica (L.) Batsch.<br>ro 2	Pirsicu	To treat gangrenous wounds, mainly when contaminated by worms or flies	Leaves	Squashed leaves are locally applied	Verb.	
Rosa canina L.<br>ro 3	Rosa 'i muntagna	Against cough	Pseudofruits (Cynhorrods)	Decoction	Verb., Orso	Decoctions of the fruits in mixture with *Malva sylvestris* L. and *Sambucus nigra* L. soothe stomach pains [6]
Rubus ulmifolius Schott<br>ro 4	Ruvine, Spine, Sirivari	Cicatrizing	Fresh leaves, as soon as picked	Squashed leaves,<br>locally applied	Buonv.,Ma.	Decoctions of the leaves are used to wash wounds [6] and as an intestinal astringent [6]. Fresh leaves are topically applied as cicatrizing means [1, 2]. The infusion of the leaves is considered emmenagogue and the decoction of the fruits diaphoretic [2]
29 Rutaceae						
Ruta graveolens L.<br>ru 1	Ruta	Anti-helmintic<br><br>To treat conjunctivitis ("gust of wind")<br><br>Headache	Leaves<br><br>Young stem with leaves	Decoction<br><br>The leaves were chewed and their essence breathed into the affected eye<br><br>The little stem was put under the hat or behind the ear	Verb., Orso.	Properties as anti-rheumatic and for treating lumbago pains are recognized to this plant [1, 6]. Moreover, the fried leaves and tops, locally rubbed, were employed for relieving pains due to inflammations at the sciatic nerve.<br>The decoction of leaves was squashed in a line wrap and the tops juice was drunk as generic analgesic. An ointment obtained frying the plant with pork fat mixed with "surfu" (= Sulphur), would be cure scabies [1]
30 Salicaceae						
Salix alba L.<br>sali 1	Salicone	Against fever and rheumatic pains	Bark	Decoction with *Sisymbrium officinale*(L.) Scop. and *Tilia platyphyllos* Scop.	Verb, Sang	
31 Scrophulariaceae						
Verbascum sp.pl.	Ntorci	To treat rheumatic	Flowers and leaves	Decoction as cataplasm or	Verb.	A macerate made of the leaves and flowers

scro 1		pains; as an haemostatic, and emollient		leaves locally applied		in olive oil has been used for similar purposes [2]. Warm compresses imbibed of the decoction of leaves are used in case of haemorrhoids; a mucilaginous decoction was drunk for treating cold and cough [6]
32 Solanaceae						
Atropa belladonna L. sola 1	not reported	Anti- arthritic and anti-rheumatic	Dried root	Powdered root mixed with pork fat	Belv. M.mo, Buonv., Gri.	The same use has been recorded in Abruzzo [43]
33 Tiliaceae						
Tilia platyphyllos Scop. tili 1	Teglia	To treat cold	Inflorescence with its bracts	Decoction with *Sisymbrium officinale*(L.) Scop., *Salix alba* L., *Malva sylvestris* L., *Althaea officinalis* L., *Mentha pulegium* L., *Eucalyptus globulus* Labill., and *Thymus pulegioides* s.l.	Verb., Sang.	
34 Umbelliferae						
Petroselium sativum Hoffm. api 1	Pitrusino, Prizzimulo	To treat toothache<br><br>To treat bee and hornet stings	Leaves<br><br>Leaves	Grinded and mixed with salt, locally applied upon affected tooth, then locally rubbed<br><br>Fresh leaves for local rubbing	Aie.	Leaves are considered laxative in children, able to stop milk flow after delivery and useful in cases of menorrhea. The poultice is considered able to treat wounds and sores, and – rubbed – to relief pains due to insect bites [6]. An infusion of the fruits is considered galactagogue
35 Urticaceae						
Parietaria officinalis L. urti 1 and *Parietaria diffusa* Mert. et Kock urti 2	Erva 'u vintu, Erva 'i muri	As a detergent for healing wounds and bruises<br><br>As an antidote against viper bite	The whole plant	Decoction from the dried plant, in washes<br><br>Decoction from the fresh plant	Verb., Orso., Gri.	In other districts of Calabria the plant is used as anti-ecchymotic [2]. Compresses made of decoction are considered lenitive for the skin inflammations and burns; crushed leaves are locally applied on bruises. The infusion is diuretic and effective in the case of renal and kidney stones. The same infusion - drunk

						for a longer period - is considered anti-rheumatic [6]. A decoction of the aerial parts, mixed with olive oil, is said to be purgative (while without oil it is considered to protect the liver [1]
Urtica dioica L. urti 3	Lurdica	Liver protecting<br>Haemorrhoids<br>Renal troubles<br>Mineralizing	Roots<br>Fresh plant<br>Leaves<br>Tops	Decoction<br>Local washing<br>The boiled leaves mixed with tomatoes were eaten to soothe renal troubles<br>Cooked (e.g. in omelettes, croquettes, and soups)	Orso., Verb., Ma.	The decoction of the leaves is an anti-hypertensive [1,2] and its is effective gastric pains;<br>in mixture with *Cynodon dactylon* (L.) Pers. and *Parietaria* spp. it is said to be diuretic [1]; an infusion of the leaves and of the roots is considered anti-anaemic, anti rachitic and able to fight the weakness [2].

Uses in italics refer to folk veterinary medicine.
[1-6] refer to uses recorded in Calabria, [7-48] to uses recorded in various Italian areas.
Abbreviation of sites: Verb = Verbicaro, Aie = Aieta, Gri = Grisolia, Buonv = Buonvicino, Ma = Maierà, Torto = Tortora, Belv.M.mo = Belvedere Marittimo, Papa = Papasidero, S. Dom.Ta = S.Domenica Talao, Orso = Orsomarso, Sang = Sangineto
